# Solitons shedding from Airy beams and bound states of breathing Airy solitons in nonlocal nonlinear media

**DOI:** 10.1038/srep09814

**Published:** 2015-04-22

**Authors:** Ming Shen, Jinsong Gao, Lijuan Ge

**Affiliations:** 1 Department of Physics, Shanghai University, 99 Shangda Road, Shanghai 200444, P. R. China; 2 School of Mathematics and Physics, Suzhou University of Science and Technology, Suzhou 215009, China

## Abstract

We investigate the spatially optical solitons shedding from Airy beams and anomalous interactions of Airy beams in nonlocal nonlinear media by means of direct numerical simulations. Numerical results show that nonlocality has profound effects on the propagation dynamics of the solitons shedding from the Airy beam. It is also shown that the strong nonlocality can support periodic intensity distribution of Airy beams with opposite bending directions. Nonlocality also provides a long-range attractive force between Airy beams, leading to the formation of stable bound states of both in-phase and out-of-phase breathing Airy solitons which always repel in local media.

Self-accelerating Airy beams have drawn considerable attention^1^,^2^,^3^ after the experimental realization^4^ of nonspreading wave packets^5^. Airy beams have many unique properties in the propagation dynamics and the applications of all-optical devices^6^,^7^,^8^,^9^,^10^,^11^,^12^,^13^. Besides the linear regime of Airy beams^6^,^7^,^8^,^9^,^10^,^11^,^12^,^13^, the nonlinear control of Airy beams also contains many novel dynamics^14^,^15^,^16^,^17^,^18^,^19^,^20^,^21^,^22^,^23^,^24^,^25^,^26^,^27^. In particular, in nonlinear media, solitons can be formed with the Airy beams^28^,^29^,^30^. Furthermore, the interactions between Airy beams^31^,^32^ play an important soul in the generation of bound soliton pairs^33^,^34^.

However, all the works mentioned above were investigated in local nonlinear media. It has been shown that the boundary conditions of a strongly nonlocal media affect deeply the propagation dynamics of self-accelerating beams^35^. Recently, an analytical expression of an Airy beam propagating in a strongly nonlocal nonlinear media was derived to show the normalized intensity distribution of the Airy beam is always periodic^36^. In optical domain, generally, nonlocal nonlinearity means that the light-induced refractive index change of a material at a particular location is determined by the light intensity in a certain neighborhood of this location. Such a nonlocal optical nonlinearity exists in nematic liquid crystals^37^ and thermal media^38^. Many works have shown that nonlocality has profound effects on the solitons propagation^39^. Nonlocal nonlinearity also affects the interaction^38^ of out-of-phase bright solitons^40^,^41^,^42^ and dark solitons^43^,^44^,^45^.

In this paper, we investigate the solitons shedding from Airy beams and anomalous interactions of Airy beams in nonlocal nonlinear media numerically. We find that nonlocality has profound effects on the propagation dynamics of the solitons shedding from Airy beam. It is also shown that the strong nonlocality can support periodic intensity distribution of Airy beams with opposite bending directions. Nonlocality also provides a long-range attractive force between Airy beams, leading to the formation of stable bound states of both in-phase and out-of-phase breathing Airy solitons which always repel in local media.

## Results

### Dynamics of solitons shedding from airy beams in nonlocal nonlinear media

We consider an Airy beam propagating in a medium with a self-focusing nonlocal cubic nonlinearity. The envelope 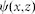 of the Airy beam is described by the normalized nonlocal nonlinear Schrodinger equation,
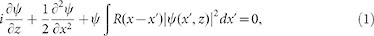
where 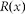 corresponds to the normalized nonlocal response function. Without loss of generality, we consider the case of so-called Gaussian nonlocal response functions^46^: 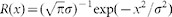, with the characteristic width 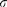 to represent the degree of nonlocality. It describes a local and a strongly nonlocal media when 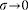 and 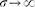
39, respectively. In general, the realistic forms of the nonlocal response functions depend on the underlying physical process of the materials^47^. It has been shown that as long as the response function is monotonically decaying, the physical properties of solitons in nonlocal media do not depend strongly on the shape of the nonlocal response function^48^. Although the Gaussian nonlocal response function is phenomenological, it can describe the general properties of other actual nonlocal media^47^.

Firstly, we study the propagation of a finite power Airy beam in nonlocal nonlinear media by considering the exponentially decaying version^6^
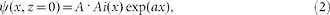
where 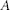 is the amplitude of the Airy beam, and 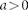 is the the decay factor to ensure containment of the infinite Airy tail and the finite power of the Airy beam^6^. For simplicity, we set 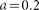 throughout this paper.

**Figure 1:**
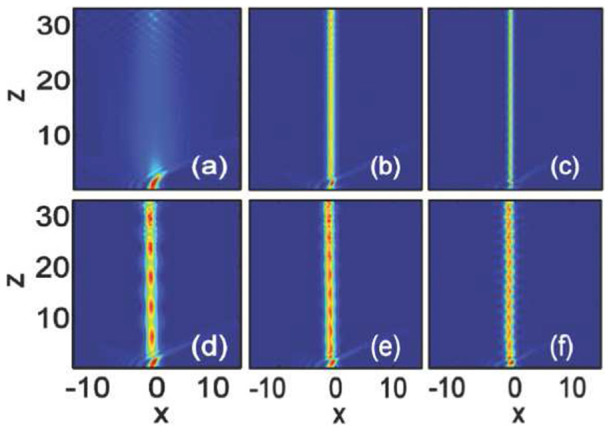
Propagation dynamics of Airy beam in nonlocal nonlinear media. The degrees of nonlocality are: (a–c) 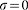 (local), and (d–f) 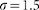. The amplitudes are: (a) 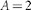, (b) 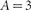, (c,d) 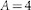, (e) 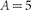, and (f) 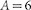.

**Figure 2:**
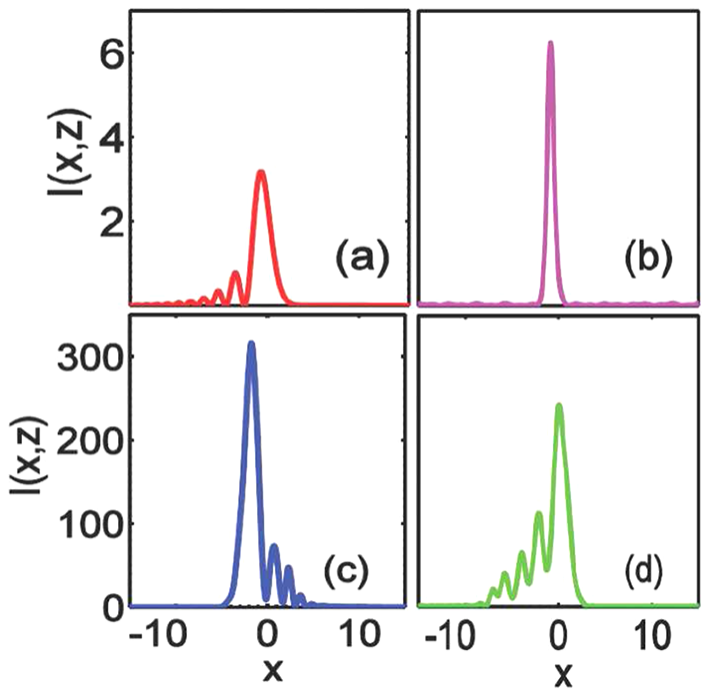
(a,b) The intensity distributions of Fig.1(c) at propagation distances 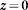 and 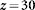 (c,d) The intensity distributions of Fig. 3(c) at propagation distances 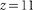 and 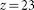.

In Fig. (1), we show the propagation dynamics of Airy beams in nonlocal nonlinear media by direct numerical integration of Eq. (1) with split-step Fourier transform method. Firstly, we consider the case of local cubic media with 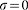 [Figs. 1(a–c)]. For small amplitude (low power), as shown in Fig. 1(a), the Airy beam performs the acceleration in space and subsequently it succumbs to diffraction. However, when 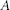 is sufficiently large^28^,^30^, a stationary soliton will be formed out of the centered energy about the Airy main lobe [Fig. 1(c)]. The intensity distribution of the Airy beam (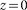) and the soliton (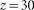) are displayed in Figs. 2(a,b). It is obvious that the peak intensity of the soliton is larger than that of the main lobe of the Airy beam, which indicates that the tail power of the Airy beam is almost confined into the main lobe to shed a soliton. The soliton exhibits periodic oscillations in the soliton amplitude and width. Similar phenomena has been studied previously^28^, which is not our aim in this paper.

If we keep the amplitude of the soliton [Fig. 1(c)] invariant, the nonlocality (e.g., 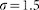) will weaken the stability of the soliton, as shown in Fig. 1(d). The period and intensity of oscillation in the soliton amplitude will become bigger and its width will become larger [Fig. 1(d)]. Although nonlocality can provide a long range attractive force to stabilize complex solitons states, it will always weaken the strength of the nonlinearity^39^. A stable soliton always require a larger amplitude with the increases of 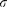
46. As shown in Figs. 1(d–f) (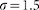), the stability of the Airy soliton is better improved when the amplitude increase, exhibited as the decrease of the beam width as well as the period and intensity of oscillation in the soliton amplitude. The intensity of the soliton keeps almost uniform for sufficiently large amplitude with snake oscillations [Fig. 1(f)]. From Figs. 1(c,f), we can also see that when a soliton is formed, the soliton will always take the main part of the initial input power, while a small fraction of the power is transformed into a self-accelerating linear packet. This phenomena is similar with the solitons dynamics obtained by using the Zakharov-Shabat scattering problem in local media^30^.

**Figure 3:**
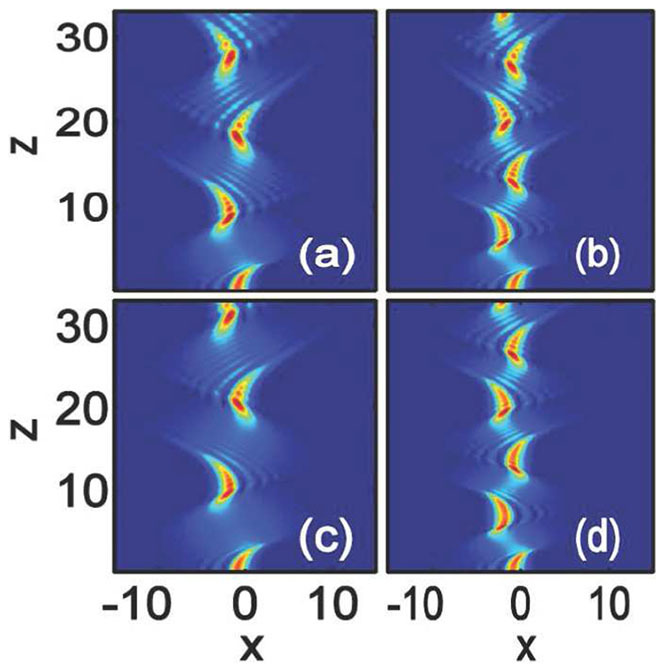
Propagation dynamics of Airy beam in strongly nonlocal nonlinear media. The degrees of nonlocality are: (a,b) 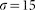, and (c,d) 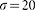. The amplitudes are: (a) 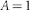, (b,c) 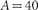, and (d) 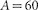.

It is also interesting that the nonlinear media cannot support stationary solitons but the periodic intensity distribution of Airy beam with opposite bending directions in strongly nonlocal regime, as shown in Fig. (3). The intensity of these beam maintain good Airy-like profile with a main lobe and the decaying tails, as shown in Figs. 2(c,d). Our numerical results of periodic intensity distribution of Airy beam obtained here agree with the analytical works of Airy beam in strongly nonlocal media very well^36^. The period will become larger when the degree of nonlocality increases or the amplitude decrease. The reason is that in the regime of strong nonlocality, the nonlocal nonlinearity trends to linear^39^ which is hard to trap the Airy beam into a soliton. However, such a strong nonlocality have a strong impact on the beam trajectory^35^: it can cause the Airy beam bending in a opposite direction periodically^36^. Besides nonlocal nonlinear media^35^,^36^, the similar phenomenon of periodic intensity distribution of Airy beam with opposite bending directions also occurs to other different physical systems, such as in plasmons with a linear refractive index profile^49^,^50^ and in curved space^51^.

### Anomalous interactions and bound states of airy solitons in nonlocal media

Next, we focus on the anomalous interactions and bound states of Airy solitons in nonlocal nonlinear media. We assume that the incident beam is composed of two shifted counter propagating Airy beams with a relative phase between them,
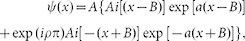
where 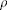 is the parameter controlling the phase shift and 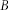 is the parameter controlling beam separations. In this report, we consider both in-phase and out-of-phase Airy beams with 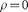 and 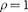, respectively^33^,^34^.

### Bound states of in-phase airy solitons

**Figure 4:**
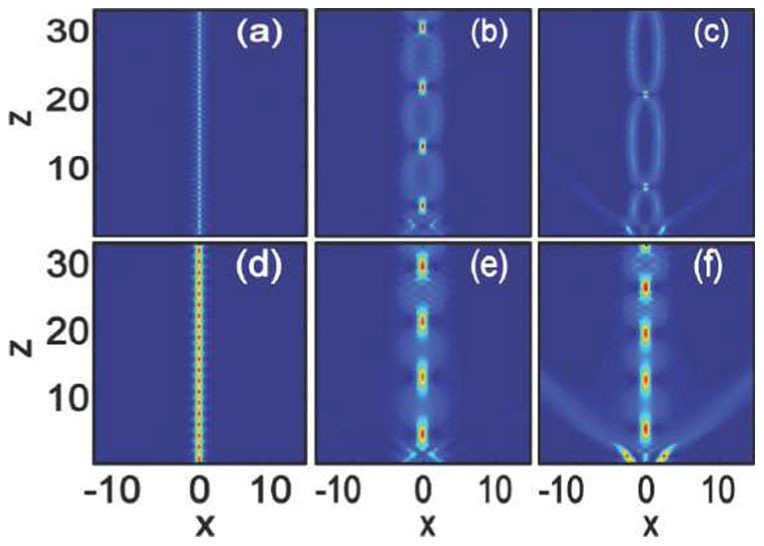
Interaction of in-phase (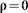) Airy beams in nonlocal nonlinear media. The degrees of nonlocality are: (a–c) 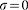 (local media), and (d–f) 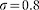. The amplitude is 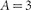 for all the plots. The beam separations are: (a,d) 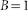, (b,e) 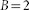, and (c,f) 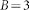.

In Fig. (4), we show the interactions between in-phase Airy beams (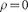) with some different beam separations. For comparison, we also re-do some previous results in local media (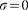) with 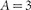
33,34. For larger separations, the two Airy components form two parallel solitons^33^,^34^, whereas, for small separations, bound breathing solitons are formed with certain periods [Figs. 4(a–c)]. The smaller the separation, the stronger the attraction and the smaller the period of soliton breathing. The attraction is the biggest when 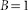 with the smallest period of the formed soliton [Fig. 4(a)]^34^. With the same amplitude 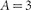, we show in Figs. 4(d–f) the interactions of the Airy beams in nonlocal nonlinear media with 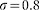. The dynamics of the interactions depend crucially on the separations of the Airy beams: for larger separations, the interactions of the Airy beams enhanced obviously [Fig. 4(f)], exhibited as the decrease of period and width, whereas, the interactions are weakened with the increase of the period and the width of the bound breathing solitons for smaller separations [Fig. 4(d)]. The nonlocality provides a long range attractive force which can enhance the interaction between solitons^52^. This is true for larger separated Airy beams [Fig. 4(f)]. For smaller separated in-phase Airy beams, the attractive force between them is big enough to form bound states with only local nonlinearity [Fig. 4(a)]. However, nonlocality will always weaken the interactions of smaller separated in-phase Airy beams [Fig. 4(d)] because of nonlocality can weaken the strength of the nonlinearity^39^.

**Figure 5:**
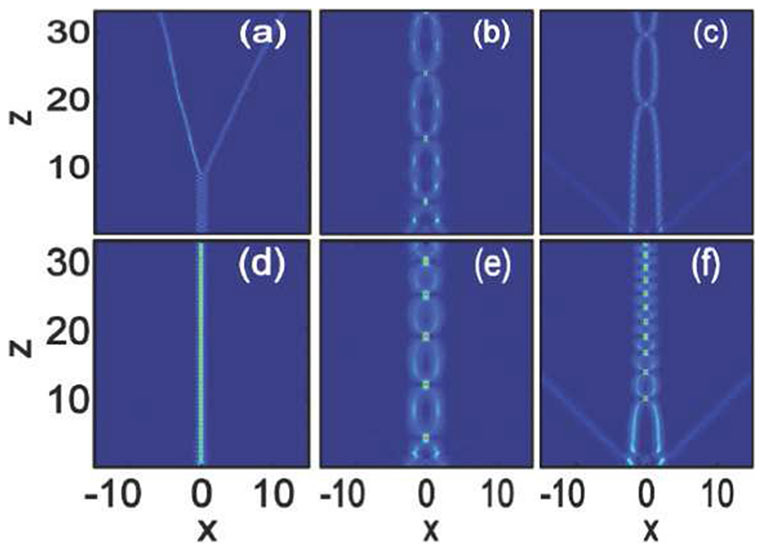
Interaction of in-phase (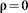) Airy beams in nonlocal nonlinear media. The degrees of nonlocality are: (a–c) 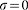 (local media), and (d–f) 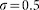. The amplitude is 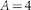 for all the plots. The beam separations are: (a,d) 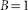, (b,e) 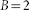, and (c,f) 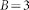.

Increasing the amplitude to 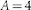, in local media, compared with the Figs. 4(a–c), the interactions are weakened obviously [Figs. 5(a,b)] except for the larger separated beam [Fig. 5(c)]. In particular, the repulsion appears for the case 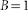, as shown in Fig. 5(a)^34^. The repulsion can be balanced by the attractive force induced even by a small degree of nonlocality. We show such bound state of breathing solitons in Fig. 5(d) with a weak nonlocality 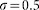. The interactions of other separated beams are also enhanced [Figs. 5(e,f)].

### Bound states of out-of-phase airy solitons

**Figure 6:**
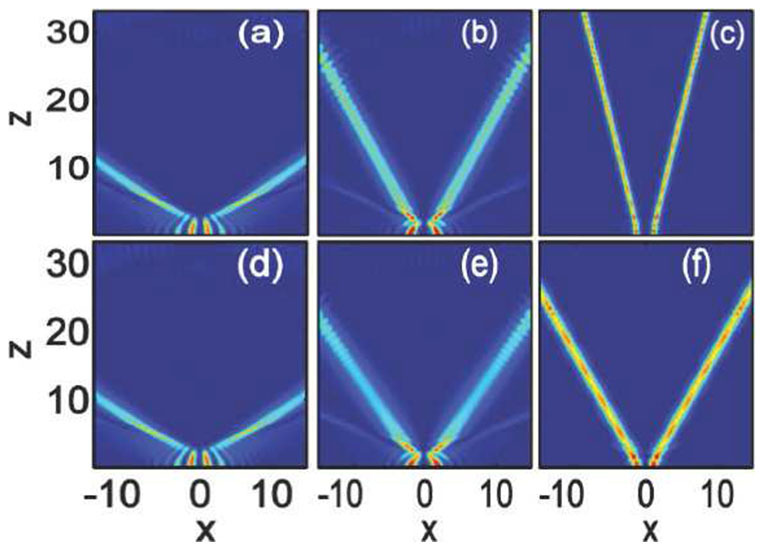
Interaction of out-of-phase (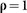) Airy beams in nonlocal nonlinear media. The degrees of nonlocality are: (a–c) 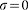 (local media), and (d–f) 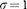. The amplitude is 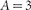 for all the plots. The beam separations are: (a,d) 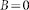, (b,e) 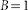, and (c,f) 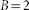.

For the out-of-phase Airy beams (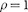), the interactions in local media and nonlocal media are shown in Figs. 6(a–c) and Figs. 6(d–f), respectively. From Figs. 6(a–c), we can see that the soliton pairs are formed from the incidence actually repel each other^34^. The smaller the interval, the stronger the repulsion. The strongest repulsion of the soliton pair happens to the case of 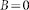 [Fig. 6(a)]^34^. For a given amplitude 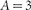, Figs. 6(d–f) clearly show the nonlocality (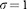) will weaken the interactions in spite of the separation is large or small due to the fact that the nonlocality cannot balance the repulsion of the out-of-phase beams.

**Figure 7:**
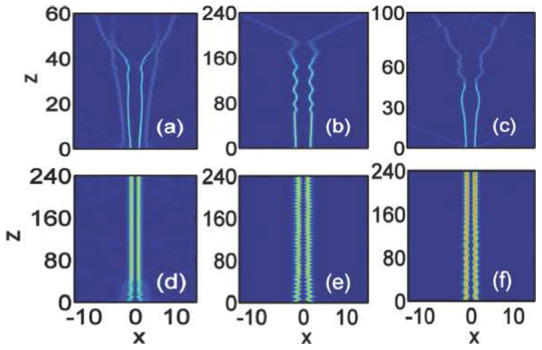
Interaction of out-of-phase (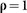) Airy beams in nonlocal nonlinear media. The degrees of nonlocality are: (a–c) 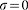 (local media), (d) 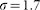, (e) 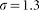, and (f) 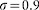. The amplitudes are 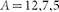 for Figs. 7(a–c) and 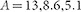 for Figs. 7(d–f), respectively. The beam separations are: (a,d) 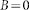, (b,e) 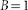, and (c,f) 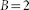.

Stationary bound states of out-of-phase Airy solitons may be obtained with larger amplitude in local media when the strong self-focusing effect balances the out-of-phase repulsion, as shown in Figs. 7(a–c). We consider the strongly repulsive case with 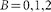. From Figs. 7(a–c), we can find that Airy beams with smaller separation always need larger amplitude to form a stable soliton pairs, e.g., 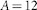 for 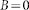 [Fig. 7(a)]. Interestingly, we can even obtain two soliton pairs with different intensities at small separation 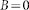 [Fig. 7(a)]. The soliton pairs cannot obtained from in-phase Airy beams with larger amplitude due to that strong self-focusing and in-phase attractive force always break up the beam, leading to the collapse of the Airy beams.

The soliton pairs obtained in local media [Figs. 7(a–c)] with large amplitude may become unstable because of the strong self-focusing effect^34^. After some propagation distances, e.g., 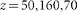 for 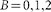, the repulsion will overtake the attraction and soliton pairs will fly away in opposite directions^34^. Completely stable out-of-phase Airy solitons bound states can be obtained with the help of nonlocality, as shown in Figs. 7(d–f). The amplitudes should also be larger than that in local media [Figs. 7(a–c)]. For such stable soliton pairs, smaller separation (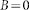) always require larger degree of nonlocality and amplitude [Fig. 7(d)]. In fact, the stable bound states of breathing soliton pairs is a result of balance between the effects of nonlocal, nonlineaity, diffraction, and repulsion. Nonlocality provides a long range attractive force to balance the repulsion of out-of-phase Airy beams, leading to the formation of bound state which always repel in local media.

## Discussions

One of the most important and interesting dynamic of the solitons is their particle-like interactions^53^. In purely local nonlinear media, the bright solitons may attract, repel, and even form bound states, depending on their relative phase^54^, whereas, the interaction of dark solitons is always repulsive^55^. Recently, the interactions of solitons have been investigated in media with spatially nonlocal nonlinearity. Nonlocal nonlinearity provides a long-range attractive force, leading to the formation of stable bound states of both out-of-phase bright solitons^40^,^41^,^42^ and dark solitons^43^,^44^,^45^. This long-range nonlocal nonlinearity also allow people to observe experimentally the multipole solitons-arrays of out-of-phase bright spots^56^.

Up to now, the interaction dynamics of Airy beam have only been investigated in local media^31^,^32^,^33^,^34^. For out-of-phase Airy beams, they are always repel in local media^33^,^34^. This repulsive force also exist in the in-phase Airy beams when their amplitudes (energy) are large enough^34^. Thus, a question arise naturally: can one obtain stable bound states (soliton pairs) of Airy beams in nonlinear media?

**Figure 8:**
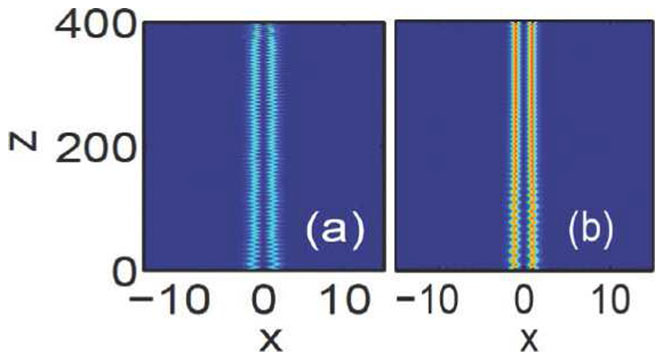
Evolution of out-of-phase (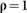) Airy beams under 5% random noise perturbations in nonlocal nonlinear media. The degrees of nonlocality are: (a) 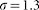, and (b) 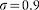. The amplitudes are 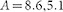 for Figs. 8(a,b), respectively. The beam separations are: (a) 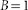, and (b) 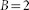.

In this report, with the help of nonlocality, we have obtained such bound states (soliton pairs) of in-phase as well as out-of-phase Airy beams in nonlocal nonlinear media. We also numerically check the stability of the bound states. We perturb the initial Airy beams by 5% random noise perturbations and then simulate their evolutions numerically. As shown in Fig. (8), we only consider the case of out-of-phase Airy beams. We can see that the bound states of breathing Airy solitons are surely stable and propagate robustly against perturbations. In summary, nonlocality provides a long-range attractive force on Airy beams, leading to the formation of stable bound states of breathing Airy solitons which always repel in local media.

## Methods

### Split-step fourier transform method

In our numerical simulations of the propagation dynamics of Airy beams and interactions of them, the split-step Fourier tansform method is used to integrate the nonlinear Schrödinger equation [Eq. (1)]. This method relies on computing the solution in small steps, and treating the linear and the nonlinear steps separately^57^. Firstly, we rewrite Eq. (1) as
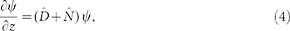
with 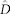 and 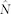 are the linear and nonlinear operator,
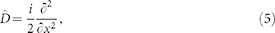

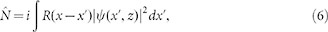
respectively. For an optical beam 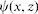 at propagation distance 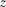, in the next step 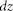, the optical field distribution 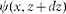 can be obtained with the Split-step method. In the first half of the step 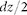, we only consider the effect of the linear operator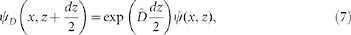 and then we consider the nonlinear operator in the whole step 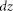



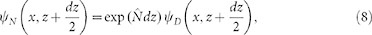
finally, in the second half of the step 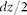, the linear operator is considered again and the optical field distribution 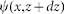 can be obtained as
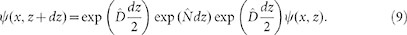


### Fourier transform of the nonlocal convolution term

In the numerical simulations with the split-step Fourier transform method, the Fourier transform of the convolution term of the nonlinear refractive index change 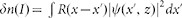 should be addressed here
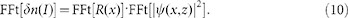


## Author Contributions

M.S. AND J.G. carried out the numerical simulations; L.G. analyzed theoretically the numerical results. Both authors wrote and reviewed the manuscript.

## References

[b1] Baumgartl J., Mazilu M. & Dholakia K. Optically mediated particle clearing using Airy wavepackets. Nat. Photon. 2, 675–678 (2008).

[b2] Polynkin P., Kolesik M., Moloney J. V., Siviloglou G. A. & Christodoulides D. N. Curved plasma channel generation using ultraintense Airy beams. Science 324, 229–232 (2009). 19359582 10.1126/science.1169544

[b3] Chong A., Renninger W. H., Christodoulides D. N. & Wise F. W. Airy-Bessel wave packets as versatile linear light bullets. Nat. Photon. 4, 103–106 (2010).

[b4] Siviloglou G. A., Broky J., Dogariu A. & Christodoulides D. N. Observation of accelerating Airy beams. Phys. Rev. Lett. 99, 213901 (2007). 18233219 10.1103/PhysRevLett.99.213901

[b5] Berry M. V. & Balazs N. L. Nonspreading wave packets. Am. J. Phys. 47, 264–267 (1979).

[b6] Siviloglou G. A. & Christodoulides D. N. Accelerating finite energy Airy beams. Opt. Lett. 32, 979–981 (2007). 17375174 10.1364/ol.32.000979

[b7] Bandres M. A. Accelerating beams. Opt. Lett. 34, 3791–3793 (2009). 20016615 10.1364/OL.34.003791

[b8] Hu Y. *et al.* Optimal control of the ballistic motion of Airy beams. Opt. Lett. 35, 2260–2262 (2010). 20596213 10.1364/OL.35.002260

[b9] Greenfield E., Segev M., Wallasik W. & Raz O. Accelerating light beams along arbitrary convex trajectories. Phys. Rev. Lett. 106, 213903 (2011). 21699298 10.1103/PhysRevLett.106.213902

[b10] Kaminer I., Bekenstein R., Nemirovsky J. & Segev M. Nondiffracting accelerating wave packets of Maxwell's equations. Phys. Rev. Lett. 108, 163901 (2012). 22680719 10.1103/PhysRevLett.108.163901

[b11] Aleahmad P. *et al.* Fully vectorial accelerating diffraction-free Helmholtz beams. Phys. Rev. Lett. 109, 203902 (2012). 23215489 10.1103/PhysRevLett.109.203902

[b12] Zhang P. *et al.* Nonparaxial Mathieu and Weber Accelerating Beams. Phys. Rev. Lett. 109, 193901 (2012). 23215384 10.1103/PhysRevLett.109.193901

[b13] Minovich A. *et al.* Generation and Near-Field Imaging of Airy Surface Plasmons. Phys. Rev. Lett. 107, 116802 (2011). 22026691 10.1103/PhysRevLett.107.116802

[b14] Ellenbogen T., Voloch-Bloch N., Ganany-Padowicz A. & Arie A. Nonlinear generation and manipulation of Airy beams. Nat. Photon. 3, 395–398 (2009).

[b15] Efremidis N. K. & Christodoulides D. N. Abruptly autofocusing waves. Opt. Lett. 35, 4045–4047 (2010). 21124607 10.1364/OL.35.004045

[b16] Hu Y. *et al.* Persistence and breakdown of Airy beams driven by an initial nonlinearity. Opt. Lett. 35, 3952–3954 (2010). 21124576 10.1364/OL.35.003952

[b17] Chen R., Yin C., Chu X. & Wang H. Effect of Kerr nonlinearity on an Airy beam. Phys. Rev. A 82, 043832 (2010).

[b18] Abdollahpour D., Suntsov S., Papazoglou D. G. & Tzortzakis S. Spatiotemporal airy light bullets in the linear and nonlinear regimes. Phys. Rev. Lett. 105, 253901 (2010). 21231591 10.1103/PhysRevLett.105.253901

[b19] Jia S., Lee J., Fleischer J. W., Siviloglou G. A. & Christodoulides D. N. Diffusion-Trapped Airy Beams in Photorefractive Media. Phys. Rev. Lett. 104, 253904 (2010). 20867382 10.1103/PhysRevLett.104.253904

[b20] Kaminer I., Segev M. & Christodoulides D. N. Self-accelerating self-trapped optical beams. Phys. Rev. Lett. 106, 213903 (2011). 21699299 10.1103/PhysRevLett.106.213903

[b21] Ament C., Polynkin P. & Moloney J. V. Supercontinuum generation with femtosecond self-healing airy pulses. Phys. Rev. Lett. 107, 243901 (2011). 22243000 10.1103/PhysRevLett.107.243901

[b22] Lotti A. *et al.* Stationary nonlinear Airy beams. Phys. Rev. A 84, 021807(R) (2011).

[b23] Dolev I., Kaminer I., Shapira A., Segev M. & Arie A. Experimental observation of self-accelerating beams in quadratic nonlinear media. Phys. Rev. Lett. 108, 113903 (2012). 22540474 10.1103/PhysRevLett.108.113903

[b24] Hu Y. *et al.* Reshaping the trajectory and spectrum of nonlinear Airy beams. Opt. Lett. 37, 3201–3203 (2012). 22859132 10.1364/OL.37.003201

[b25] Zhang P. *et al.* Generation of linear and nonlinear nonparaxial accelerating beams. Opt. Lett. 37, 2820–2822 (2012). 22825145 10.1364/OL.37.002820

[b26] Driben R. & Meier T. Nonlinear dynamics of Airy-Vortex 3D wave packets: Emission of vortex light waves. Opt. Lett. 39, 5539–5542 (2014). 25360922 10.1364/OL.39.005539

[b27] Chen R., Chew K. -H. & He, S. Dynamic control of collapse in a vortex Airy beam. Sci. Rep. 3, 1406; 10.1038/srep01406 (2013). 23518858 PMC3604801

[b28] Fattal Y., Rudnick A. & Marom D. M. Soliton shedding from Airy pulses in Kerr media. Opt. Express 19, 17298–17307 (2011). 21935094 10.1364/OE.19.017298

[b29] Driben R., Konotop V. V. & Meier T. Coupled Airy breathers. Opt. Lett. 39, 5523–5526 (2014). 25360918 10.1364/OL.39.005523

[b30] Allayarov I. M. & Tsoy E. N. Dynamics of Airy beams in nonlinear media. Phys. Rev. A 90, 023852 (2014).

[b31] Rudnick A. & Marom D. M. Airy-soliton interactions in Kerr media. Opt. Express 19, 25570–25582 (2011). 22273950 10.1364/OE.19.025570

[b32] Wiersma N., Marsal N., Sciamanna M. & Wolfersberger D. All-optical interconnects using Airy beams. Opt. Lett. 39, 5997–6000 (2014). 25361139 10.1364/OL.39.005997

[b33] Zhang Y. *et al.* Soliton pair generation in the interactions of airy and nonlinear accelerating beams. Opt. Lett. 38, 4585–4588 (2013). 24322080 10.1364/OL.38.004585

[b34] Zhang Y. *et al.* Interactions of Airy beams, nonlinear accelerating beams, and induced solitons in Kerr and saturable nonlinear media. Opt. Express 22, 7160–7171 (2014). 24664064 10.1364/OE.22.007160

[b35] Bekenstein R. & Segev M. Self-accelerating optical beams in highly nonlocal nonlinear media. Opt. Express 19, 23706–23715 (2011). 22109397 10.1364/OE.19.023706

[b36] Zhou G., Chen R. & Ru G. Propagation of an Airy beam in a strongly nonlocal nonlinear media. Laser Phys. Lett. 11 105001 (2014).

[b37] Peccianti M., Conti C., Assanto G., Luca A. D. & Umeton C. Routing of anisotropic spatial solitons and modulational instability in liquid crystals. Nature (London) 432, 733–737 (2004). 15592407 10.1038/nature03101

[b38] Rotschild C., Alfassi B., Cohen O. & Segev M. Long-range interactions between optical solitons. Nat. Phys. 2, 769–774 (2006).

[b39] Krolikowski W. *et al.* Modulational instability, solitons and beam propagation in spatially nonlocal nonlinear media. J. Opt. B: Quantum Semiclass. Opt. 6, S288 (2004).

[b40] Rasmussen P. D., Bang O. & Krolikowski W. Theory of nonlocal soliton interaction in nematic liquid crystals. Phys. Rev. E 72, 066611 (2005). 10.1103/PhysRevE.72.066611 16486082

[b41] Peccianti M., Brzdakiewicz K. & Assanto G. Nonlocal spatial soliton interactions in nematic liquid crystals. Opt. Lett. 27, 1460–1462 (2002). 18026479 10.1364/ol.27.001460

[b42] Hu W., Zhang T., Guo Q., Xuan L. & Lan S. Nonlocality-controlled interaction of spatial solitons in nematic liquid crystals. Appl. Phys. Lett. 89, 071111 (2006).

[b43] Nikolov N. *et al.* Attraction of nonlocal dark optical solitons. Opt. Lett. 29, 286–288 (2004). 14759053 10.1364/ol.29.000286

[b44] Kong Q., Wang Q., Bang O. & Krolikowski W. Analytical theory for the dark-soliton interaction in nonlocal nonlinear materials with an arbitrary degree of nonlocality. Phys. Rev. A 82, 013826 (2010).

[b45] Dreischuh A., Neshev D. N., Petersen D. E., Bang O. & Krolikowski W. Observation of attraction between dark solitons. Phys. Rev. Lett. 96, 043901 (2006). 16486823 10.1103/PhysRevLett.96.043901

[b46] Buccoliero D., Desyatnikov A. S., Krolikowski W. & Kivshar Y. S. Laguerre and Hermite Soliton Clusters in Nonlocal Nonlinear Media. Phys. Rev. Lett. 98, 053901 (2007). 17358858 10.1103/PhysRevLett.98.053901

[b47] Wyller J., Krolikowski W., Bang O. & Rasmussen J. J. Generic features of modulational instability in nonlocal Kerr media. Phys. Rev. E 66, 066615 (2002). 10.1103/PhysRevE.66.066615 12513437

[b48] Zhong W. & Belic M. Three-dimensional optical vortex and necklace solitons in highly nonlocal nonlinear media. Phys. Rev. A 79, 023804 (2009).

[b49] Zhang P. *et al.* Plasmonic Airy beams with dynamically controlled trajectories. Opt. Lett. 36, 3191–3193 (2011). 21847204 10.1364/OL.36.003191

[b50] Liu W., Neshev D. N., Shadrivov I. V., Miroshnichenko A. E. & Kivshar Y. S. Plasmonic Airy beam manipulation in linear optical potentials. Opt. Lett. 36, 1164–1166 (2011). 21479017 10.1364/OL.36.001164

[b51] Bekenstein R., Nemirovsky J., Kaminer I. & Segev M. Shape-Preserving Accelerating Electromagnetic Wave Packets in Curved Space. Phys. Rev. X 4, 011038 (2014).

[b52] Chen W. *et al.* Interactions of nonlocal dark solitons under competing cubic-quintic nonlinearities. Opt. Lett. 39, 1764–1767 (2014). 24686599 10.1364/OL.39.001764

[b53] Stegeman G. I. & Segev M. Optical Spatial Solitons and Their Interactions: Universality and Diversity. Science 286, 1518–1523 (1999). 10567250 10.1126/science.286.5444.1518

[b54] Krolikowski W., Saffman M., Luther-Davies B. & Denz C. Anomalous Interaction of Spatial Solitons in Photorefractive Media. Phys. Rev. Lett. 80, 3240–3243 (1998).

[b55] Zhao W. & Bourkoff E. Interactions between dark solitons. Opt. Lett. 14, 1371–1373 (1989). 19759686 10.1364/ol.14.001371

[b56] Rotschild C. *et al.* Two-dimensional multi-pole solitons in nonlocal nonlinear media. Opt. Lett. 31, 3312–3314 (2006). 17072407 10.1364/ol.31.003312

[b57] Agrawal G. P. Nonlinear fiber optics (Academic Press, San Diego, 1995).

